# Inbreeding and Genetic Erosion from a Finite Model of a Synthetic Formed with Single Crosses

**DOI:** 10.3390/plants12030541

**Published:** 2023-01-25

**Authors:** Jaime Sahagún-Castellanos, Aureliano Peña-Lomelí, Denise Arellano-Suarez, Juan Enrique Rodríguez-Pérez

**Affiliations:** Departamento de Fitotecnia, Universidad Autónoma Chapingo (UACh), Chapingo 56230, Mexico

**Keywords:** *Zea mays* L., genotypic array, random mating, diallelic experiment, hybrid

## Abstract

When a seed produced by a single-cross (SC) maize hybrid is sown, the resulting grain yield is usually lower than that of the hybrid due to the inbreeding generated. However, if a seed from a mixture of *s* hybrids were sown instead, the synthetic variety thus formed (*Syn_SC_*) would have a lower inbreeding coefficient (*FSyn_SC_*) and a higher grain yield. The grain yield *s*, the finite number of representatives of each parent SC (*m*) and the inbreeding coefficient of the parent lines of the SCs (*F*) are related to the *FSyn_SC_*. In addition, randomness and the finite size of *m* can cause the loss of genes and genotypes and increase the *FSyn_SC_*. The objectives of this study were to derive formulas for (1) expressing *FSyn_SC_* in terms of *m*, *F*, and *s*, and (2) calculating the probability of the occurrence of gene and genotype loss. It was found that for the probability of no genotype being missing from the progeny representing a parent to be at least 0.95, it is necessary that *m* ≥ 15. It was also found that a sample size of 7 is sufficient for *FSyn_SC_* to stabilize, more visibly as *F* is larger, and for the probability of the occurrence of erosion to be practically zero.

## 1. Introduction

Synthetic varieties (SVs) of maize (*Zea mays* L.) are usually highly heterogeneous and heterozygous populations that adapt even to unfavorable environmental conditions. For example, Farid et al. [1] worked on the breeding of maize in drought conditions and developed 2 SVs that yielded at least 6.0 t ha^−1^. On the other hand, Badu-Apraku et al. [2], after five selection cycles of *S*_1_ families for drought resistance from a SV, obtained a genetic gain of 423 kg ha^−1^ cycle^−1^.

However, since these varieties resulted from the random mating of a population of finite size, they may include the formation of progenies generated by mating between related individuals. This type of progeny, however, usually has a reduction in vigor [3,4]. For example, it can result in reduced seed yield in onions (*Allium cepa* L.) [5] and reduced grain yield in maize [6].

Commonly, a maize SV originates from the random mating of a set of selected lines. However, it is not common for good-quality lines to be available for the development of SVs oriented towards farmers who are unlikely to have the economic ability to regularly acquire seeds of hybrid varieties. Sometimes, when farmers sow a hybrid variety in one growing season, in the next season, they sow seeds produced by that variety, with a consequent reduction in the means of variables such as grain yield due to the inbreeding produced in one generation.

This effect of inbreeding could be attenuated if, instead of sowing seeds harvested from a single hybrid, the seeds harvested from a hybrid produced by sowing a mixture of two or more varieties of this type were sown. Villanueva et al. [7], from the results of a diallelic experiment on nine commercial hybrids, found that two crosses between hybrids could be successfully used as SVs. The idea of generating a SV whose parents are several hybrids has generated interest, along with the need to conduct the research necessary to answer the questions that arise in this context.

The relationship between lines, hybrids and SVs has been studied for a long time. According to Wright [8]’s formula, the behavior of each synthetic that can be formed with *p* lines can be estimated with the data from a complete diallelic experiment comprising those *p* lines. The estimation of the behavior of any synthetic formed by a subset of *p*′ lines (*p*′ ≤ *p*) is conducted with the experimental data of the *p*′ lines and their *p*′(*p*′ − 1) single crosses. On the other hand, the open-pollinated varieties developed by the International Maize and Wheat Improvement Center (CIMMYT) for their yield potential and tolerance of adverse biotic and abiotic factors are actually synthetics whose parents are lines resulting from the process of hybrid-variety development [9].

The information generated by a diallelic experiment, in addition to helping to predict the yield of each SV that could possibly be formed with the *p*′ lines, can contribute to determining the optimal number of parents that a synthetic variety should have; moreover, it allows the identification of single crosses of good quality and outstanding parents per se [10].

The information required in terms of the optimal number of lines for the development of synthetic varieties is not, however, the only useful information of this type. Until recently, little attention was paid to the question concerning the number of plants that should represent each parent of a synthetic variety [11,12].

This number (*m*) is important because it can affect the inbreeding coefficient and the probability of gene loss in the synthetic variety. On this topic, the most common approach has been to proceed as if the number of representative plants used were of a sufficient size, so that the frequencies of the progeny of each parent were in accordance with the Hardy–Weinberg law. The difficulty that arises in this context is how to properly interpret what “sufficient size” means.

Evidently, the inbreeding coefficient (IC) and the stability of the genotypic array of a population that reproduces by random mating in successive generations can be affected by the randomness of the genetic mechanism operating during gametogenesis when the number of representatives of each parent is finite and their genotypes are heterozygous.

In a study on a SV formed by *s* single crosses (*Syn_SC_*) that in turn were generated with unrelated 2*s* lines and an inbreeding coefficient equal to *F*, Escalante-González et al. [11] considered that each parent is represented by *m* plants and that the number of non-identical-by-descent (NIBD) genes that they contain is a random variable. To this end, the authors derived its mean and variance. However, they did not study the relationship of the number of representatives of each *Syn_SC_* parent (*m*) with the probabilities of gene and genotype loss and the magnitude of the *Syn_SC_* inbreeding coefficient (*FSyn_SC_*). In the context of the Hardy–Weinberg law, there must be a sample size of the progeny of each parent in which changes in the *Syn_SC_* inbreeding coefficient and the probability of genotype and gene loss are negligible. The objectives of this research were to (1) derive the *FSyn_SC_* in terms of *m*, *s* and *F*, and (2) calculate the probability of losing one or more genes and genotypes when each parent is represented by a finite number of plants. With this information, the aim is to determine the minimum sample size necessary for *FSyn_SC_* to stabilize and for the probability of losing one or two genes to be negligible.

## 2. Results

### 2.1. Inbreeding Coefficient

Consider the model:

*m* = 4*x* + *e*

where *x* = 0, 1, 2, 3, … and *e* = 0, 1, 2, 3.

In this model, *m* is the sample size of the progeny of each single cross, *x* is the largest positive integer such that 4*x* ≤ *m*, and *e* is the number of plants that failed to form a complete group of four (*e* = 1, 2, 3). The synthetic variety whose parents are *s* single crosses (*Syn_SC_*) was visualized as the population generated by the random mating of the *ms* plants representing the *s* parents. As this mating in turn involves the random mating of any subset of the *ms* plants, particularly of the *m* plants of a single cross, the derivation of the inbreeding coefficient of *Syn_SC_* (*FSyn_SC_*) was based on the ICs of the progenies produced by the random mating of the *m* plants of a parent. This mating in turn involves the random mating of each complete group of four plants whose genotypes are those that form the genotypic array generated by the single cross. Consequently, if the lines that are parents of the single cross are A_1_A_2_ and B_1_B_2_, the sampled population is formed by the genotypes of the genotypic array produced by the single cross A_1_A_2_ × B_1_B_2_ (GEA). This is:

(1) $$\mathrm{GEA}=\left(\frac{1}{4}\right)A_{1}B_{1}+\left(\frac{1}{4}\right)A_{1}B_{2}+\left(\frac{1}{4}\right)A_{2}B_{1}+\left(\frac{1}{4}\right)A_{2}B_{2}$$

The basic results used to derive the formula for *FSyn_SC_* were obtained from the progenies produced by the random mating of the genotypes that form the GEA (Equation (1)). These results are shown in Table 1. In this derivation, the inbreeding of the progeny produced by the random mating of these four plants was considered to have two sources: (1) four selfings and (2) 4 × 3 = 12 intragroup crosses, of which six were direct and six were reciprocal (Table 1).

The contributions to inbreeding by the selfings and intragroup crosses were, on average, 1/2 and (1 + 2*F*)/6, respectively (Table 1). The following also contributed to inbreeding: (a) the 16*x*(*x* − 1) crosses between each of the plants in a group of four with each of those in another group of four from the same parent (the results can be presented in complete tables, as in Table 1); and (b) selfings and crosses involving the *e* plants that failed to complete a group of four. The latter included *e*(*e* − 1) intragroup crosses, both direct and reciprocal, along with the 8*xe* intergroup crosses of the *e* plants that failed to complete a group with the 8*x* plants from the complete groups, 4*x* direct and 4*x* reciprocal. The selfings of these *e* plants were included in those of the *m* plants representing a parent. The above considerations regarding *FSyn_SC_* allowed us to arrive at the following formula:

(2) $$FSyn_{sc}=\frac{m(1/2)+\left[12x+e\left(e-1\right)\right]\frac{\left(1+2F\right)}{6}+\left[16x\left(x-1\right)+8xe\right]\frac{\left(1+F\right)}{4}}{m^{2}s}$$

m=4x+e;   0≤F≤1;    e=0, 1, 2, 3;   x=0, 1, 2, 3,…

Obviously, the formula for *FSyn_SC_* (Equation (2)) is general. It can be used to determine the exact value of *FSyn_SC_* for any combination of values of *m*, *F* and *s*. It is the only formula that has this quality. The previously derived formulas for the IC of a *Syn_SC_* [13] correspond to “large samples”.

The magnitude and significance of the precision with which the inbreeding coefficient is expressed must be considered. In the development of a SV, in addition to offering objective guidance for determining the sample size (*m*) of each parent, these factors are related to the means of quantitative characters of economic importance. In maize, for example, they relate to grain yield, stubble yield, etc. [6].

Based on Equation (2), the inbreeding coefficients of the progeny of a single cross were calculated for the 120 combinations of 24 values of *m* (1, 2, 3, …, 24) and 5 of *F* (0.000, 0.500, 0.750, 0.875, and 1.000). The results are shown in Table 2. Among these, the following stand out:

Whenever *m* is a multiple of 4 (4, 8, 12, 16, …), the inbreeding coefficients of the progeny produced by the random mating of the *m* plants are equal to 0.250, 0.375, 0.4375, 0.4687, and 0.5000 when the inbreeding coefficients of the parent lines of the single cross (*F*) are 0.000, 0.500, 0.750, 0.875, and 1.000, respectively. Furthermore, as the value of *m* increases, the values of *FSyn_SC_* tend towards those obtained when *m* is a multiple of 4, increasing in speed as the value of *F* increases.

For *x* = 1, 2, …, 6, the values of *FSyn_SC_* corresponding to sample sizes 4*x* + 1, 4*x* + 2 and 4*x* + 3 are greater than those of 4*x* and 5*x*, but decrease as the value of *m* increases. In addition, whenever *m* = 1 or *F* = 1, *FSyn_SC_* = 0.5000.

Whenever *m* > 1, a positive relationship between the values of *F* and those of *FSyn_SC_* is observed.

The explanation for the results in Table 2 is obvious in some cases. For example, when *m* = 1, the “intragroup” progeny reproduces through the fertilization of a single plant whose genotype is made up of two NIBD genes and whose progeny must have an IC equal to 0.5, regardless of the value of *F*. Moreover, when *F* = 1, each parent single cross is represented by *m* plants with the same genotype formed by two NIBD genes. Thus, intragroup random mating generates a progeny whose genotypic array is the same as that produced by a single plant by selfing and which, consequently, has an IC equal to 0.5, regardless of the value of *m.*

The number of sets of frequencies (SFs) with which the GEA genotypes in (Equation (1)) appear in a sample is a function of *m*[*f*(*m*)]. Subsequently, the *k*-th SF [*k* = 1, 2, …, *f*(*m*)] is formed by the frequencies fk1m, fk2m,fk3m, and fk4m for genotypes A_1_B_1_, A_1_B_2_, A_2_B_1_ and A_2_B_2_, respectively. Furthermore, the number of ways in which this *k*-th SF can occur in the sample of size m [(NF)km] must be:

(3) (NF)km=m!(fk1m!)(fk2m!)(fk3m!)(fk4m!) ; ∑i=14fkim=m ;  k=1,2, …f(m)   

### 2.2. Probability of Inclusion of GEA Genotypes (Equation (1)) in the Sample

Unfortunately, the probability that the genotypes contained in a sample size of four are all four in the GEA (Equation (1)) is very low: 4!/4^4^ = 0.094. With larger sample sizes, the probability that all four genotypes are included must be increased. For example, with *m* = 5, the number of equally possible and mutually exclusive ways in which the sample includes all four genotypes, with one of them repeated once, is [5!/2!1!1!1!]4 = 240, while the total number of equally possible and mutually exclusive outcomes is 4^5^ = 1024. Therefore, with *m* = 5, the probability that the sample contains all four genotypes is 240/1024 = 0.234. This probability is also too low.

To calculate the probability that the sample would include all four GEA genotypes in (Equation (1)) for larger sample sizes, the methods and elements used by Rodriguez-Perez et al. [14] were adapted to this case.

If the number of different permutations (*NP*) in which the four frequencies of the *k*-th SF can be assigned to the four genotypes that make up the sample is (NP)km, and if Pklm is the number of times that the *l*-th smallest frequency (*l* = 1, 2, 3, 4) of the *k*-th SF appears, then:

(4) (NP)km=4!(Pk1m!)(Pk2m!)(Pk3m!)(Pk4m!) k=1,2,…, f(m) 

Evidently, the total number of equally possible and mutually exclusive events generated by sampling with replacement of size *m* (*TN*) is 4*^m^*, that is:

*TN* = 4*^m^* (5)

According to Equations (3)–(5), the formulas for calculating the probability that the size *m* sample contains all four GEA genotypes in (Equation (1)) according to the *k*-th SF [*P_k_* (GEA inclusion)*_m_*] and for all SFs are, respectively:

(6) Pk(GEA inclusion)m=[(NF)km][(NP)km]4m            k=1,2,…f(m)

and

(7) ∑k=1f(m)Pk(GEA inclusion)m=∑k=1f(m)[(NF)km][(NP)km]4m

For example, based on Equation (6), the probabilities were generated for each of the three possible SFs, so that with *m* = 7, all four GEA genotypes were included. First, in this case, the SFs are: *k* = (1): 1, 1, 1, 4; *k* = (2): 1, 2, 2, 2; and *k* = (3): 1, 1, 2, 3. The number of different permutations was calculated for each SF [(NP)k7]. For example, in SF *k* = (3), the total number of different permutations was 4!/(2!1!1!0!) = 12. These are shown in Table 3.

The calculation of the probability that a sample of seven would include the four genotypes of the genotypic array of the cross A_1_A_2_ × B_1_B_2_ was based on Equations (6) and (7) and is presented in Table 4. This probability was 0.513, but this was not satisfactory. A sample size that is compatible with the standards of certainty typically required by plant breeders must provide a probability of at least 0.95.

In the search for a size compatible with acceptable reliability, the probability that all four GEA genotypes would be included in samples of 8 and 12 was calculated (Equation (1)). These probabilities were 0.62 and 0.70, respectively. It was at *m* = 15 that, based on Equation (7), the probability of inclusion of the four GEA genotypes was found to reach the value of 0.95.

### 2.3. Probability of Gene Loss

When the sample of a single cross is generated, one or two genes can be lost. Two are lost when the *m* plants that make up the sample have the same genotype. The loss of one gene occurs only when the sample contains one genotype *v* times (*v* = 1, 2, …, *m* − 1) and another in the remaining times (*m* ≥ 2), except when the two genotypes are: (1) A_1_B_1_ and A_2_B_2_ or (2) A_1_B_2_ and A_2_B_1_. In these two cases, there is no gene loss.

In a hypothetical case in which *m* = 1, the probability of losing two genes is 100%. If *m* = 2, the loss of two genes occurs when the two plants that make up the sample have the same genotype; this event can occur in four ways, each with a probability of (1/4)^2^. This means that when *m* = 2, the probability of losing two genes is 4(1/4)^2^ = 0.25. In general, the probability that with a sample size *m* two genes are lost (*_m_P*_2_) is:

(*_m_P*_2_) = 4(1/4)*^m^* (8)

If *m* = 2, the loss of one gene can also occur. This occurred in each of the four samples (Table 5).

To calculate the probability of losing one gene when *m* = 2(_1,1;2_*P*_1_), one must consider that each of the four samples (Table 5) can occur in two orders and that the total number of equally possible and mutually exclusive events is 4^2^. Therefore, with *m* = 2, _1,1;2_*P*_1_ = (2 × 4)/4^2^ = 0.5.

Hereafter, the general expression *_v_*_,*m*−*v*;*m*_*P*_1_ is used to represent the probability that a sample of size *m* contains *v* plants that have the same genotype (*v* = 1, 2, …, *m* − 1) and *m* − *v* another and that both genotypes lack the same gene of the four samples in GEA (Equation (1)). The formula is:

(9) Pv,m−v;m1=m!v!(m−v)!4m

With *m* = 3, the loss of one gene can occur in eight ways. The origin of these can be visualized as if in each of the four samples of two in which a gene is lost (Table 5), one of the two genotypes is duplicated in one case and the other in the second case. For example, from sample (A_1_B_1_,A_1_B_2_), the two visualized samples, now of size three, are: (1) A_1_B_1_, A_1_B_1_, A_1_B_2_ = (2A_1_B_1_, A_1_B_2_) and (2) A_1_B_1_, A_1_B_2_, A_1_B_2_ = (A_1_B_1_, 2A_1_B_2_). Therefore, since each of the four samples of two lacking the same gene (Table 5) is associated with two size samples of three lacking the same gene, the probability that one gene is lost when *m* = 3 in each of the eight cases (P2,1;31) is as in (Equation (9)): [3!/(2! 1!)]/4^3^ = 0.046875. The total probability of the eight cases is 8 [0.046875] = 0.375.

Regarding the probability that one gene is lost when *m* = 4 (P41), the three samples of that size associated with the samples of two lacking the A_2_ gene were samples 1, 2 and 3, as shown in Table 6. Samples 1 and 3 have the same probability of occurrence  (P3,1;41), which differs from that of sample 2 (P2,2;41). Based on Equation (9) and the data in Table 6:

P41=8[P3,1;41]+4[P2,2;41]=8[4!/(3!1!)]/44+4[4!/(2!2!)]/44=0.219

The formulas for calculating the probability of losing one gene in samples of size *m* ≥ 3 (*_m_P*_1*e*_ and *_m_P*_1*o*_) are:

(10) Pm1e=8∑v=1m2−1Pm−v,v;m1o+4[Pm2,m2;m1]  if m is even and m≥4

and

(11) Pm1o=8∑v=1m−12Pm−v,v;m1  if m is odd and m≥3             

Based on Equations (9)–(11), Table 7, containing the probabilities of one-gene (*_m_P*_1_), two-gene (*_m_P*_2_), and one- or two-gene (*_m_P*_1,2_) losses for twelve sample sizes was completed.

With the exception of when *m* = 1, the loss of one gene is more likely than that of two (Table 7). This is because while the loss of two genes occurs only when the sample only includes one genotype of the four in the GEA (Equation (1)) *m* times, the loss of one gene requires that, for example, the sample of the form *v*A_1_B_1_ and (*m* − *v*)A_1_B_2_ does not include A_2_ and has *m* − 1 ways of being constructed (*v* = 1, 2, …, *m* − 1), each of which has *m*!/[(*m* − *v*)!*v*!] different orders of occurrence. There are three other cases of this kind. These result when A_1_, B_1_ or B_2_ is lost.

## 3. Discussion

It was found that for each of the five *F* values studied combined with each of the *m* values that are multiples of 4 (*m* = 4*x*, for *x* = 1, 2, 3, 4, 5, 6), the *FSyn_SC_* values are equal (Table 2). This result is due to the fact that with these *m* values, the expected frequencies of the genotypes that make up the sample are equal (1/4). Furthermore, for the three values of *m* (4*x* + 1, 4*x* + 2 and 4*x* + 3; *x* = 1, 2, 3, 4, 5, 6) that are between two numbers of *m* that are consecutive multiples of 4 [between 4*x* and (4*x* + 4); *x* = 1, 2, 3, 4, 5, 6], the ICs are greater than those of the two that are consecutive multiples of 4. This occurs because for these three values of *FSyn_SC_*, the frequencies of the four genotypes of the GEA (Equation (1)) are not equal and, in these cases, matings between plants with the same genotype are more frequent and generate more inbreeding than when genotypic frequencies are equal [15]. When genotypic frequencies are equal, the mating frequencies between different genotypes that contribute less to inbreeding are maximized.

It is also noticeable (as shown in Table 2) that as *m* grows, the *FSyn_SC_* values corresponding to the three sample sizes that are between two consecutive multiples of 4 are increasingly close to these sizes [4*x* and 5*x*]. This trend is related to the decrease in the variance of the number of NIBD genes in the SCs that occurs when *m* is larger. This variance, [2*^m^*^+2^ − 8][1 − *F*]^2^/4*^m^* [11], also decreases as *F* increases.

It is noteworthy that the smallest *FSyn_SC_* values for each value of *F* occur when *m* = 4, 8, 12, 16 (Table 2). This result is to be expected according to Equation (2). This reduces to the form *FSyn_SC_* = (1 + *F*)/4 whenever *m* is a multiple of 4. On the other hand, although the use of small *m* values is desirable, the decision to use a size of *m* = 4, for example, would not be appropriate without taking into account other results. For example, it is also desirable for the sample to include all four of the GEA genotypes in (Equation (1)), which means that the entire gene pool of the parents can be involved in the formation of the *Syn_SC_*. However, the probabilities of sample sizes of four, eight, and twelve including all four GEA genotypes are not satisfactory. According to the calculation for *m* = 4 and the results from Equation (7), these probabilities are 0.094, 0.623, and 0.8747, respectively. In addition, it was found that: (1) a sample of 15 is required for the probability that all four GEA genotypes included in (Equation (1)) to be 0.95, and (2) from *m* = 15, *FSyn_SC_* is practically unchanged and becomes more stable as the value of *F* increases (Table 2) and approaches (1 + *F*)/4. It should be considered, however, that a sample that does not include one or two of the four genotypes may contain all four GEA genes (Equation (1)) and, therefore, may avoid the loss of genes and genotypes in the *Syn_SC_*. For example, if a sample only contains the genotypes A_1_B_1_, A_1_B_2_, and A_2_B_1_, its gametic array will include all four genes, A_1_, A_2_, B_1_, and B_2_. With these genes in the gametic array, the random mating of the sample should form a genotypic array that includes all four genotypes of the single-cross A_1_A_2_ × B_1_B_2_. This result is also produced by a sample containing only the genotypes A_1_B_2_ and A_2_B_1_.

If the gametic array of the sample representing a single cross is missing one or two of the four genes, the resulting genotypic array was not complete. This loss of genes, unlike that of the genotypes, is always irreparable and results in increased *Syn_SC_* inbreeding. Fortunately, the probability of loss is not high (Table 7). In the case of losing one gene from *m* = 7 onwards, this probability is negligible and for the loss of two genes, the probability of occurrence is also negligible (Table 7), even from a sample of five.

## 4. Materials and Methods

Due to the characteristics of maize (*Zea mays* L.), the model on which this study was based was that of a locus of a diploid species that reproduces by random mating. The parents of the SV under study, *s* single crosses, were considered to be formed with unrelated 2*s* lines whose inbreeding coefficient was *F* (0 ≤ *F* ≤ 1). This means that if a line that is a parent in a single cross is represented as A_1_A_2_, the probability (P) that A_1_ and A_2_ are identical by descent (≡) is *F*; that is, P(A_1_ ≡ A_2_) = *F* [16]. In a similar way, it could be argued that each of the 2*s* lines are parents of the *s* single crosses. This, in turn, means that when the genes A_1_ and A_2_ are not identical by descent (100 (1 − *F*) 100% of the cases) they must be alleles. It should be noted that in all cases, A_1_ and A_2_ were referred to as genes.

According to the characteristics of their origin, the genotypes of the plants representing a SV parent were visualized as those captured by a random sample of size *m* taken with replacement from the population formed by the genotypes produced by a single cross.

The derivation of the formula for *FSyn_SC_* was based on the consideration that the random mating of the *ms* plants representing the *s* parent single crosses of *Syn_SC_* implies the random mating of any subset of *ms* plants, particularly the *m* representatives of each single cross. This intraparental mating is the only source of inbreeding in *Syn_SC_*. All other matings are interparental crosses. These do not contribute to inbreeding, since the lines are unrelated. For this reason, the derivation of *FSyn_SC_* in terms of a finite number *m* was based on the IC of the progeny of *m* plants from a single cross. This derivation was conducted based on the procedure proposed by Rodríguez-Pérez et al. [17].

To investigate how sample size affects *FSyn_SC_*, the IC of all combinations of 24 values of *m* with 5 ICs of the parent lines of a single cross was calculated. The calculation of the probability that the sample of *m* plants of a single cross includes all the genotypes that form the genotypic array and that 1 or 2 genes are lost was also performed for different values of *m*.

## 5. Conclusions

A formula was derived to determine the inbreeding coefficient of a synthetic variety developed by the random mating of *s* single crosses formed by unrelated 2*s* lines whose inbreeding coefficient is *F* (*FSyn_SC_*). Unlike previously derived formulas for this synthetic, *FSyn_SC_* can be applied to any number combination of representatives of each parent (*m*), number of parent single crosses (*s*), and inbreeding coefficient of the lines (*F*).

The greatest effect of the finite *m* size on *FSyn_SC_* occurs at values from 1 to 4. In addition, whenever *m* is a multiple of 4, the *FSyn_SC_* values corresponding to the same *F* value do not differ and their value is the smallest. This result is consistent with what would be expected for the genotypic array of a “large” population reproduced by random mating. Regarding the rest of the *m* values, very small reductions in *FSyn_SC_* are observed from *m* = 7 and tend towards an *m* value that is a multiple of 4.

It was also found that for the probability of the sample including all four genotypes being at least 0.95, it is necessary that *m* ≥ 15. However, in an apparent contradiction, it was found that the probability of the sample not including one or two genes is practically negligible from *m* = 7. This means that since all four genes are included in the sample, all four GEA genotypes must be present in the *Syn_SC_* (Equation (1)) and *FSyn_SC_* could be closer to its smallest value.

## Figures and Tables

**Table 1:** Average inbreeding coefficients of progenies produced by random mating of a group of 4 plants whose genotypes are those generated by the single-cross A_1_A_2_ × B_1_B_2_, (Equation (1)). The inbreeding coefficient of the lines is *F* and they are unrelated.

Genotypes	Genotypes				Mean
	A_1_B_1_	A_1_B_2_	A_2_B_1_	A_2_B_2_	
A_1_B_1_	1/2	(1 + *F*)/4	(1 + *F*)/4	*F*/2	(1 + *F*)/4
A_1_B_2_	(1 + *F*)/4	1/2	*F*/2	(1 + *F*)/4	(1 + *F*)/4
A_2_B_1_	(1 + *F*)/4	*F*/2	1/2	(1 + *F*)/4	(1 + *F*)/4
A_2_B_2_	*F*/2	(1 + *F*)/4	(1 + *F*)/4	1/2	(1 + *F*)/4
Mean	(1 + *F*)/4	(1 + *F*)/4	(1 + *F*)/4	(1 + *F*)/4	(1 + *F*)/4

**Table 2:** Inbreeding coefficients of the progenies produced by random mating of the *m* representatives of a single cross. The parent lines of the cross are unrelated and their inbreeding coefficient is equal to *F*. The calculation was performed with the formula of Equation (2) for *FSyn_SC_* with *s* = 1.

*m*	*F*				
	0.0000	0.5000	0.7500	0.8750	1.0000
1	0.5000	0.5000	0.5000	0.5000	0.5000
2	0.3333	0.4167	0.4583	0.4792	0.5000
3	0.2778	0.3889	0.4444	0.4722	0.5000
4	0.2500	0.3750	0.4375	0.4688	0.5000
5	0.2600	0.3800	0.4400	0.4700	0.5000
6	0.2593	0.3796	0.4398	0.4699	0.5000
7	0.2551	0.3776	0.4388	0.4694	0.5000
8	0.2500	0.3750	0.4375	0.4688	0.5000
9	0.2531	0.3765	0.4383	0.4691	0.5000
10	0.2533	0.3767	0.4383	0.4692	0.5000
11	0.2521	0.3760	0.4380	0.4690	0.5000
12	0.2500	0.3750	0.4375	0.4688	0.5000
13	0.2515	0.3757	0.4379	0.4689	0.5000
14	0.2517	0.3759	0.4379	0.4690	0.5000
15	0.2511	0.3756	0.4378	0.4689	0.5000
16	0.2500	0.3750	0.4375	0.4688	0.5000
17	0.2509	0.3754	0.4377	0.4689	0.5000
18	0.2510	0.3755	0.4378	0.4689	0.5000
19	0.2507	0.3753	0.4377	0.4688	0.5000
20	0.2500	0.3750	0.4375	0.4688	0.5000
21	0.2506	0.3753	0.4376	0.4688	0.5000
22	0.2507	0.3753	0.4377	0.4688	0.5000
23	0.2505	0.3752	0.4376	0.4688	0.5000
24	0.2500	0.3750	0.4375	0.4688	0.5000

To obtain the *FSyn_SC_* values, the ICs in Table 2 are multiplied by (1/*s*).

**Table 3:** Different permutations of the frequencies of SF *k* = (3): 1, 1, 2, 3 of the genotypes that can be produced by the single-cross A_1_A_2_ × B_1_B_2_ when the progeny consists of 7 plants (*m* = 7).

Permutations	Genotypes			
	A_1_B_1_	A_1_B_2_	A_2_B_1_	A_2_B_2_
1	1	1	2	3
2	1	1	3	2
3	1	2	1	3
4	1	2	3	1
5	1	3	1	2
6	1	3	2	1
7	2	1	1	3
8	2	1	3	1
9	2	3	1	1
10	3	1	1	2
11	3	1	2	1
12	3	2	1	1

**Table 4:** Probability that with *m* = 7. a sample would include all four GEA genotypes (Equation (1)). The result of the calculation of the number of different permutations of each of the 3 possible SFs [(NP)k7], and of the probability of occurrence of the genotypes according to each SF [(NF)k7/47].

k	Genotypes				(NP)k7	(NF)k7/47	$P_{k}{\left(\mathrm{GEA}\;\mathrm{Inclusion}\right)}_{7}$
	A_1_B_1_	A_1_B_2_	A_2_B_1_	A_2_B_2_			
1	1	1	1	4	4	210/4^7^	0.05127
2	1	2	2	2	4	630/4^7^	0.15381
3	1	1	2	3	12	420/4^7^	0.30761
$\overset{3}{\underset{k=1}{\sum }}P_{k}{\left(\mathrm{GEA}\;\mathrm{inclusion}\right)}_{7}=$							0.51269

Only one permutation of the genotypic frequencies is shown for each of the three sets of frequencies (*k* = 1, 2, 3).

**Table 5:** GEA (Equation (1)) for samples of two lacking one gene.

Number	1	2	3	4
**Sample**	A_1_B_1_ A_1_B_2_	A_1_B_1_ A_2_B_1_	A_1_B_2_ A_2_B_2_	A_2_B_1_ A_2_B_2_

**Table 6:** GEA in (Equation (1)) for samples of four lacking one gene.

Num.	Sample	Num.	Sample	Num.	Sample	Num.	Sample
1	3A_1_B_1_, A_1_B_2_	4	3A_2_B_1_, A_2_B_2_	7	3A_1_B_2_, A_2_B_2_	10	3A_2_B_1_, A_2_B_2_
2	2A_1_B_1_, 2A_1_B_2_	5	2A_2_B_1_, 2A_2_B_2_	8	2A_1_B_2_, 2A_2_B_2_	11	2A_2_B_1_, 2A_2_B_2_
3	A_1_B_1_, 3A_1_B_2_	6	A_2_B_1_, 3A_2_B_2_	9	A_1_B_2_, 3A_2_B_2_	12	A_2_B_1_, 3A_2_B_2_

**Table 7:** Probabilities of loss (PL) of one gene (*_m_P*_1_), of two genes *_m_P*_2_, and of one or two genes (*_m_P*_1,2_) in the formation of the progeny of size *m* from a parent single cross of a *Syn_SC_*.

PL	*m*											
	1	2	3	4	5	6	7	8	9	10	11	12
*_m_P*_1_	0.000	0.500	0.375	0.227	0.118	0.061	0.037	0.016	0.009	0.004	0.002	0.000
*_m_P*_2_	1.000	0.250	0.063	0.016	0.004	0.001	0.010	0.000	0.000	0.000	0.000	0.000
*_m_P*_1,2_	1.000	0.750	0.438	0.235	0.122	0.062	0.047	0.016	0.009	0.004	0.002	0.000

## Data Availability

Not applicable.
